# Discovery by the Late Horace Wells of the Applicability of Nitrous Oxyd Gas, Sulphuric Ether and Other Vapors, in Surgical Operations

**Published:** 1853-01

**Authors:** 


*Discovery by the late Horace Wells of the Applicability of Nitrous Oxyd Gas, Sulphuric Ether and other Vapors, in Surgical Operations, nearly two years before the Patented Discovery of Drs. Charles T. Jackson and W. G. Morton.* Hartford, 1852.

This is the title of a pamphlet which has been lying for some time upon our table. The ether controversy bids fair to be as interminable as the contention between the Sonnites and Shiites, and productive of about as much benefit to the world at large. The history of the invention is a plain one and multiplication of words cannot change it. Every chemist knew the intoxicating property of these various gases and vapors, and the possibility of applying this to the treatment of surgical diseases, seems, as is usual in these practical applications of scientific facts, to have occurred to several persons at once. The pamphlet makes out a strong case for Dr. Wells; but, whatever may have been the time of the discovery of the possibility of using these agents for the purposes for which they are now employed, Morton certainly deserves whatever credit attaches to the actual introduction of them to the notice of the public.

